# Out-of-hospital cardiac arrest patients treated with cardiopulmonary resuscitation using extracorporeal membrane oxygenation: focus on survival rate and neurologic outcome

**DOI:** 10.1186/s13049-016-0266-8

**Published:** 2016-05-18

**Authors:** Jae Jun Lee, Sang Jin Han, Hyoung Soo Kim, Kyung Soon Hong, Hyun Hee Choi, Kyu Tae Park, Jeong Yeol Seo, Tae Hun Lee, Heung Cheol Kim, Seonju Kim, Sun Hee Lee, Sung Mi Hwang, Sang Ook Ha

**Affiliations:** Department of Anesthesiology, Hallym University Medical Center, Chuncheon, South Korea; Division of Cardiology, Department of Internal Medicine, Hallym University Medical Center, Chuncheon, South Korea; Department of Thoracic and Cardiovascular Surgery, Hallym University Medical Center Sacred Heart Hospital, 22 Gwanpyeong-ro 170 beon-gil, Dongan-gu, Anyang-si, Gyeonggi-do 431-070 South Korea; Department of Emergency Medicine, Hallym University Medical Center, Chuncheon, South Korea; Department of Radiology, Hallym University Medical Center, Chuncheon, South Korea

**Keywords:** Extracorporeal membrane oxygenation, Neurologic outcome, Oliguria, Out-of-hospital cardiac arrest, Survival rate

## Abstract

**Background:**

Extracorporeal membrane oxygenation (ECMO) is a useful treatment for refractory out-of-hospital cardiac arrest (OHCA). However, little is known about the predictors of survival and neurologic outcome after ECMO. We analyzed our institution’s experience with ECMO for refractory OHCA and evaluated the predictors of survival and neurologic outcome after ECMO.

**Methods:**

This was a retrospective review of the medical records of 23 patients who were treated with ECMO due to OHCA that was unresponsive to conventional cardiopulmonary resuscitation, between January 2009 and January 2014.

**Results:**

Our ECMO team was activated within 10 min for refractory OHCA, and the 30-day survival rate was 43.5 %. In a multivariate analysis that evaluated independent factors contributing to mortality, urine output  ≤ 0.5 mL · kg^−1^ · h^−1^ (defined as oliguria) during the 24 h after ECMO was statistically significant (OR, 32.271; 95 % CI, 1.379–755.282; *p* = 0.031). Just after ECMO implantation, 6 of the 9 patients (66.7 %) who had normal findings on brain computed tomography (CT) survived with a cerebral performance category (CPC) of grade 1. However, only 3 of the 11 patients (27 %) who had evidence of hypoxic brain damage on initial brain CT survived (their CPC grade was 4).

**Conclusions:**

Based on our findings, the survival rate can be improved by rapid implantation of ECMO, and oliguria seen during the first 24 h after ECMO may be an independent predictor of mortality. Furthermore, findings on brain CT just after ECMO and subsequent images may represent an important predictor for neurologic outcome after ECMO.

## Background

Out-of-hospital cardiac arrest (OHCA) has a poor prognosis, with survival rates between 4 and 39.3 % [1–5]. Cardiac arrest patients can tolerate only a short period of circulatory disturbance and the chances of survival decrease rapidly when cardiopulmonary resuscitation (CPR) lasts over 15–30 min [6, 7]. Furthermore, refractory cardiac arrest, defined as persistent circulatory failure despite more than 30 min of appropriate CPR, is usually fatal in the intensive care unit [8].

Extracorporeal membrane oxygenation (ECMO) is an aggressive and invasive type of extracorporeal life support (ECLS) that has been suggested for refractory cardiac arrest [9]. ECMO can be performed during resuscitation, and it provides sufficient perfusion of vital organs during the treatment of cardiac arrest and provides injured myocardium with the chance to recover [10]. Several recent studies have shown favorable outcomes regarding survival after in-hospital cardiac arrest and in OHCA patients receiving ECLS [1, 4, 5, 11]. However, few reports have analyzed its effectiveness in terms of neurological outcomes in OHCA patients.

The present study retrospectively evaluated our institution’s results with ECMO in adult patients with refractory OHCA. This study focused on survival rate, neurological outcomes as indicated by brain imaging findings, and prognostic indicators.

## Methods

### Patients

**Fig. 1:**
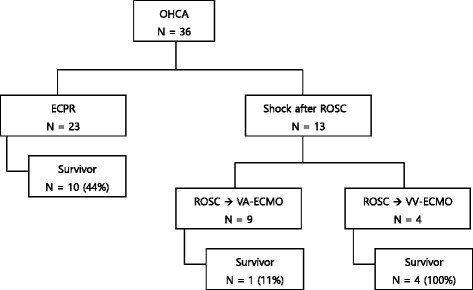
Flow diagram of the study population and outcome ECMO, extracorporeal membrane oxygenation; OHCA, out-of-hospital; ECPR, extracorporeal cardiopulmonary resuscitation; VA, venoarterial; VV, venovenous

This study received approval from our institutional review board (IRB No. 2012–93) Hallym University Medical Center, Chuncheon. Informed consent was waived due to its retrospective study design. Of 119 patients who underwent ECMO between January 2009 and January 2014, this study retrospectively reviewed the records of 23 patients who had cardiac arrest outside of the hospital but did not achieve return of spontaneous circulation (ROSC) within 10 min of CPR, or patients with recurrent arrests despite ROSC for over 20 min within 2 h after arriving at the hospital (Fig. 1). In addition, patients with ongoing intracranial hemorrhage or terminal malignancy, those who required constant support, and those who underwent unwitnessed cardiac arrest were excluded from the study. All patients with OHCA were treated by the emergency medical technician (EMT), who trained for basic life support (BLS). At the scene, EMT performed 30 chest compression and 2 bag valve mask ventilation. Especially, automated external defibrillator was applied for a patient with shockable rhythm. After 10 cycles of BLS at the scene, the patient was transported to our emergency department by ambulance, thereafter emergency medical staff performed advanced life support.

After arriving at the hospital, the subjects received CPR under emergency medicine staff supervision. If ROSC was not achieved within 10 min of beginning CPR, the ECMO team reassessed each subject’s status. If the status indicated ECMO, the ECMO was immediately implanted in the catheterization laboratory during cardiac compressions.

### ECMO equipment and management

Three types of centrifugal pumps were used to deliver the ECMO: the Capiox Emergency Bypass System® (Terumo, Inc., Tokyo, Japan) and the Bio-pump® (Medtronic Inc., Minneapolis, MN, USA) were used from January 2009 to May 2010; from June 2010 onward, a Centrifugal Rotaflow pump® (Maquet Inc., Hirrlingen, Germany) was used in most patients. Depending on patient size, we used 17–21 Fr arterial cannulae (DLP®, Bio-Medicus, Medtronic Inc.; or RMI®, Edwards Lifesciences LLC, Irvine, CA, USA) and 17–28 Fr venous cannulae (DLP®, Bio-Medicus, Medtronic Inc.; or RMI®, Edwards Lifesciences LLC).

ECMO was performed in the Catheterization Lab with an injection of heparin at 50–80 u/kg, followed by fluoroscopy guided cannulation of the femoral artery and vein via the Seldinger method during cardiac compression. Our center used an anticoagulant, nafamostat mesilate (SK Chemicals Life Science Biz., Seoul, Korea Licensed by Torii Pharmaceutical Co. Ltd, Tokyo, Japan) at 0.4–1.5 mg · kg^−1^ · h^−1^ and maintained the partial thromboplastin time at 60–80 s to decrease the bleeding risk caused by ECMO. Patients who did not have a high bleeding risk were maintained with heparin injections of 300–1,400 u/h to achieve an activated clotting time of 140–180 s.

After ECMO, patients in whom coronary artery disease was suspected underwent coronary angiography and received percutaneous coronary intervention. Intra-aortic balloon pump was taken in patients who had no improvement in cardiogenic shock despite high doses of inotropics before ECMO, or patients who had a mean blood pressure below 40 mmHg despite the use of inotropics after ECMO. Brain computed tomography (CT) was checked after ECMO began and all procedures were performed in the catheterization laboratory when the Glasgow coma score (GCS) was below 7 (eyes open: to pain, motor: localized pain, verbal: intubated state) [12]. Then CT and magnetic resonance imaging (MRI) of the brain were then performed and the CT was checked in cases of 1) GCS below 7 during the 72 h after ECMO, or 2) change in pupil size with an absent pupil light reflex in patients who recovered the reflex. MRI was used to evaluate brain function of surviving patients before discharge. The criteria for the hypoxic brain damage in the brain CT was 1) Diffuse mass effect with effacement of the cerebral sulci and of the brainstem cisterns 2) Global decrease in the cortical gray-matter density from edema, causing loss of the normal gray-white matter differentiation 3) Low density lesions of the basal ganglia bilaterally 4) Decreased gray matter density in the watershed distributions bilaterally. Especially, gray/white matter ratio was an en early predictor for hypoxic brain damage [13, 14]. Hypothermia therapy was performed when the GCS was below 9 (eyes open: to speech, motor: obeys, verbal: intubated state) after arrival to the intensive care unit. This was provided using a heater unit (Maquet Inc., Hirrlingen, Germany) by maintaining the body temperature at 33–34 °C for 24 h without sedatives. If the GCS rose above 9, the patient’s body temperature was raised 0.5 °C per hour using sedatives.

The ECMO flow was maintained at 3.0–4.0 L/min, while the mean blood pressure was sustained at >60 mmHg. If necessary, norepinephrine or dopamine was also administered. For patients who received percutaneous coronary intervention (PCI), clopidogrel 300 mg and aspirin 250 mg were administered on the date of the procedure; from the second day onward, the doses were reduced to 75 mg and 100–200 mg, respectively. Care was taken to maintain the hematocrit level and platelet count at 30–35 % and 50,000–80,000/μL, respectively, and a transfusion was given when these values decreased. A 2D-echo was done daily to check for thrombi in the heart chambers and to ensure improvement of wall motion. ECMO was removed when the ejection fraction exceeded 30 % at the ECMO flow of 1 L/min on echocardiography. Successful ECMO weaning and survival were defined as cases of survival > 24 h after the removal of ECMO and > 30 d after the ECMO run.

### Statistical analysis

Statistical analyses were performed using the IBM SPSS Statistics program (ver. 21; IBM Co., NY, USA); continuous and categorical variables were analyzed using the Mann Whitney *U*-test and the Pearson chi square test or Fisher’s exact test, respectively. The univariate and multivariate stepwise logistic regression analysis model was used to identify independent mortality-related factors. Values of *p* < 0.05 were considered statistically significant. Survival outcomes of the patients who survived OHCA were analyzed using the Kaplan-Meier survival method.

## Results

Totally 36 patients with OHCA were transported to emergency room. Among these patients, 13 patients had archived ROSC after conventional CPR. The four drowning patients received venovenous ECMO due to refractory hypoxia after ROSC. These patients were 30 day survivors and discharged with CPC 1 score. Nine patients received venoarternial ECMO due to shock after ROSC. Only one patient was a 30 day survivor (Fig. 1). In the case of E-CPR, AMI (15 cases) was the most common cause, followed by anaphylaxis (bee sting 2 cases, drug allergy 1 case), ventricular fibrillation (2 cases), commotion cordis (assault 1 case), hypothermia (1 case, falling in a river for 34 minutes in the winter) and AAA rupture (1 case).

The clinical characteristics of the survival group and mortality group are compared in Table 1. The patients’ age, gender, body mass index, pre-ECMO sequential organ failure assessment, simplified acute physiology score 2 score, the use of an intra-aortic balloon pump (IABP), PCI, and continuous renal replacement therapy showed no statistical significance, but PaO2 in the pre-ECMO laboratory findings was statistically significant (*p* = 0.009).

**Table 1:** Comparison of patients’ clinical characteristics between non-survivors and survivors

	All	Non-survivors	Survivors	*p*
	*N* = 23	*N* = 13	*N* = 10	
Age, years	55 (40, 68)	57 (46.5, 72.5)	52 (35.5, 61)	0.208
Gender, male (%)	20	10	10	0.229
BMI	22.8 (21.9, 26.1)	22.9 (21.2, 28.1)	22.6 (22.4, 24.1)	0.648
Past medical history				
Hypertension	12	8	4	0.414
Diabetes	12	8	4	0.414
Previous PCI	4	3	1	0.604
Cerebral vascular accident	2	1	1	1.000
Causes of cardiac arrest				0.329
AMI	15	8	7	
Anaphylactic shock	3	3	-	
Arrhythmia	2	1	1	
Commotio Cordis	1	-	1	
Hypothermia	1	-	1	
AAA rupture	1	1	-	
Pre-ECMO laboratory findings				
pH	7.01 (6.80, 7.08)	6.98 (6.80, 7.13)	7.02 (6.97, 7.04)	0.422
PaO2	33 (7, 54.5)	10 (6, 42)	51 (28, 70)	0.009^a^
PaCO2	69 (55.5, 97.5)	71 (64.5, 103)	61 (37.5, 88)	0.095
CK-MB	6.1 (2.2, 15.73)	4.4 (2.0, 24.2)	6.1 (2.5, 7.6)	0.976
Troponin-I	0.17 (0.06, 3.25)	0.17 (0.07, 6.15)	0.17 (0.05, 1.03)	0.522
BUN	14.5 (11.3, 20.1)	14.5 (12.1, 21.0)	16.0 (9.2, 20.5)	0.927
Creatinine	1.2 (1.1, 1.4)	1.3 (0.8, 1.4)	1.2 (1.1, 1.5)	0.784
Total bilirubin	0.78 (0.59, 1.13)	0.75 (0.57, 1.11)	0.92 (0.7, 1.21)	0.410
Lactate	9.7 (7.7, 13.2)	10.4 (8.4, 13.9)	9.0 (6.9, 11.6)	0.284
Pre-ECMO				
SOFA score	14 (13, 16)	14 (13.5, 16)	14 (12.8, 15.3)	0.446
SAPS 2 score	87 (80, 97)	90 (83.5, 99.5)	82.5 (75, 91.3)	0.077
PCI	15 (65.2 %)	9 (69.2 %)	6 (60 %)	0.646
IABP				0.604
Pre-ECMO	2	1	1	
After-ECMO	2	2	-	
CRRT	18	10	8	1.000

*BMI* body mass index, *PCI* percutaneous coronary intervention, *AAA* abdominal aortic aneurysm, *BUN* blood urea nitrogen, *SOFA*, sequential organ failure assessment, *SAPS 2* simplified acute physiology score, *IABP* intra-aortic balloon pump, *CRRT* continuous renal replacement therapy, *IQR* continuous variables expressed median

^a^significant difference

Most patients (63 %) experienced cardiac arrest in public, and were given CPR after an average of 1 min. Fourteen patients (61 %) were given CPR by a bystander. The median time from collapse to arrival at hospital was 22 min, and 87 % of the patients showed a rhythm of Vf or VT at arrival. Only three patients achieved ROSC after CPR at the hospital and they were maintained for over 20 min, but eventually went through ECMO because of recurrent cardiac arrest. The median door to ECMO time was 55 min (*p* = 0.030). A positive pupil light reflex (*p* = 0.007), vasoactive inotropic score after 6 h of ECMO (*p* = 0.030), 24 h urine output (<0.5 mL · kg^−1^ · h^−1^) (*p* < 0.001) after admission, and ECMO maintenance time (*p* = 0.008) showed statistical significance (Table 2) [15]. CT of the brain was performed just after ECMO implantation in 20/23 patients (87 %). Three patients were excluded, because one who survived had a GCS of 9 and the other two patients, who died, could not be maintained on ECMO flow due to abdominal aortic rupture and massive gastrointestinal bleeding, respectively. On the initial brain CT, 9 patients showed normal findings but 11 had evidence of hypoxic brain damage. In the follow-up brain images, 3 surviving patients had normal findings, 3 had evidence of mild hypoxic brain damage with a CPC grade of 1, and 3 had evidence of hypoxic brain damage of CPC grade 4 (Table 3). The complications related to CPR and ECMO are shown in Table 4.

**Table 2:** Comparison of CPR- and ECMO-related characteristics between non-survivors and survivors

	All	Non-survivors	Survivors	*p*
	*N* = 23	*N* = 13	*N* = 10	
Locations of victims				0.472
Public	15	10	5	
Home	5	2	3	
In-ambulance during transportation	2	1	1	
Other hospital	1	-	1	
Time of collapse to CPR start, min	1 (1, 5)	4 (1, 6)	1 (1, 1.75)	0.166
Bystander CPR	14	6	8	0.197
First monitored rhythm				0.685
Shockable	10	5	5	
Non-shockable	13	8	5	
Transport time from collapse to hospital, min	22 (15, 38)	22 (15, 51)	21 (13, 30)	0.910
EKG rhythm on arrival at ED				0.177
Vf/VT	20	12	8	
PEA	2	-	2	
Asystole	1	1	-	
Collapse to ECMO time, min	84 (61, 101)	94 (72.5, 127)	64.5 (55.25, 89.75)	0.101
CPR time, min	62 (53, 86)	66 (58.5, 108)	57.5 (40.5, 73.75)	0.101
ROSC ≥ 20 min during CPR	3	2	1	1.000
Door to ECMO time, min	55 (41, 67)	62 (49, 71.5)	45 (32.5, 56.5)	0.030^a^
ROSB at ICU arrival	17	10	7	1.000
Pre-ECMO VIS	41.0 (16.4, 76.2)	63.5 (0, 93.8)	39.6 (31.7, 71.6)	0.832
Post-ECMO VIS; at 6 h on ICU arrival	7.6 (0, 22.6)	11.3 (3.4, 58.4)	0 (0, 7.8)	0.030^a^
24 h urine output (ml · kg^−1^ · h^−1^)	0.92 (0.03, 2.33)	0.1 (0–0.92)	2.39 (1.59, 3.86)	<0.001^a^
Pupil diameter on arrival at ED, mm	6 (6, 7)	7 (6, 7)	6 (3.5, 7.25)	0.414
Pupil reflex positive after ECMO	15	6	10	0.007^a^
Hypothermia for 24 h	18	12	6	0.127
ECMO duration, h	98 (60, 192)	75 (18.5, 122)	151 (97.25,219.25)	0.008^a^
CPC grade				
1			7	
4			3	

VIS (vasoactive inotropic score) = IS + milrinone dose (μg/kg/min) + 10000 × vasopressin dose (unit/kg/min) + 100 × norepinephrine dose (μg/kg/min), IS (inotropic score) = dopamine dose (μg/kg/min) + dobutamine dose (μg/kg/min) + 100 × epinephrine dose (μg/kg/min)

*CPR* cardiopulmonary resuscitation, *ECMO* extracorporeal membrane oxygenation, *ED* emergency department, *Vf* ventricular fibrillation, *VT* ventricular tachycardia, *PEA* pulseless electrical activity, *ROSC* return of spontaneous circulation, *ROSB* return of spontaneous beat, *ICU* intensive care unit, *CPC* cerebral performance category, *IQR* continuous variables expressed median

^a^significant difference

**Table 3:** Characteristics of brain images

Brain CT just after ECMO	FU brain image	Outcomes		Total
		Death	Survival	
		*N* = 13	*N* = 10	*N* = 23
Hypoxic brain damage (−) *n* = 9	Hypoxic brain damage (−)	-	3	3
	Hypoxic brain damage (+)	1	3	4
	ICH due to hypoxic brain damage	2	-	2
Hypoxic brain damage (+) *n* = 11	Hypoxic brain damage (+)	6	3	9
	ICH due to hypoxic brain damage	2	-	2
Check (−) *n* = 3	Check (−)	2	1	3

*CT* computer tomography, *ICH* intracranial hemorrhage

**Table 4:** Comparison of complications between non-survivors and survivors

	All (N-=23)	Non-survivors (N=13)	Survivors (N=10)	*p*
CPR-related complication				
Chest wall compartment syndrome	1	1	-	1.000
Pneumothorax	2	1	1	1.000
Chylothorax	1	-	1	0.435
Pulmonary hemorrhage	2	2	-	0.486
Hypoxic brain damage	14	11	3	0.001^a^
Cannula related complication				
Cannula site bleeding, ≥ 2 units/d pRBC transfusion	3	3	-	0.229
Leg ischemia d/t septic emboli and right common iliac artery obstruction	2	-	2	0.178
Acute renal failure	11	6	5	1.000
Ulcer bleeding	1	1	-	1.000
Pneumonia	15	5	10	0.003^a^
ICH d/t hypoxic brain damage	4	4	-	0.104
Sore	4	-	4	0.024^a^
Sepsis	5	2	3	0.618

*CPR* cardiopulmonary resuscitation, *d* day, *pRBC* packed red blood cell, *ICH* intracranial hemorrhage

^a^significant difference

**Fig. 2:**
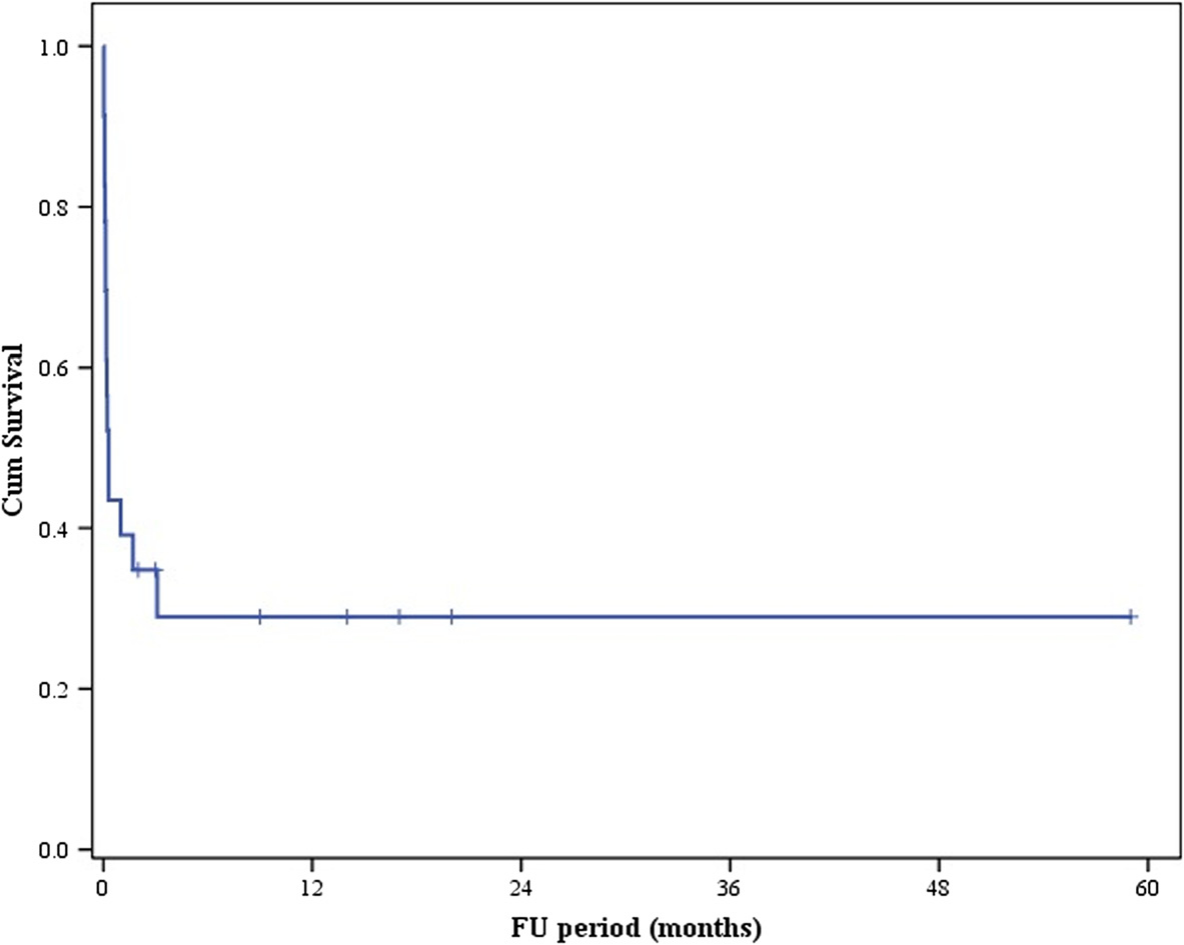
Kaplan-Meier survival curve. All seven patients considered fit for discharge survived

In a univariate analysis evaluating independent factors of mortality, the pre-ECMO PaO2 (odds ratio [OR], 0.946; 95 % confidence interval [CI], 0.901–0.993; *p* = 0.025), and a 24 h urine output of ≤0.5 mL · kg^−1^ · h^−1^ (OR, 20.250; 95 % CI, 11.878–218.390; *p* = 0.013) showed statistical significance. However, in multivariate analysis, only the 24 h urine output of ≤ 0.5 mL · kg^−1^ · h^−1^ (OR, 32.271; 95 % CI, 1.379–755.282; *p* = 0.031) showed statistical significance (Table 5). Of the 10 survivors, 3 patients died on the 38^th^, 50^th^, and 94^th^ day in the hospital, respectively, and of the 7 patients who were discharged, 6 had a CPC of grade 1 and 1 had a CPC of grade 4, all of whom survived without any complications for an average of 19 months (range, 1–57 months) (Fig. 2).

**Table 5:** Univariate analysis and multiple logistic regression analysis of predictors for mortality

Variables	Univariate analysis			Multivariate analysis		
	OR	95 % CI	*p*	OR	95 % CI	*p*
Pre-ECMO PaO2	0.946	0.901–0.993	0.025^a^	0.936	0.873–1.005	0.068
24 h urine output ≤ 0.5 mL · kg^−1^ · h^−1^	20.250	1.878–218.390	0.013^a^	32.271	1.379–755.282	0.031^a^

*ECMO* extracorporeal membrane oxygenation, *OR* odds ratio, *CI* confidence interval.

^a^significant difference

## Discussion

Prolonged CPR is associated with poor outcomes, and only a small percentage of patients return to their former lives without complications [6–8, 16, 17]. In order to overcome this limitation, E-CPR was considered as an alternative treatment for cardiopulmonary arrest. In animal model, Trummer et al. demonstrated that E-PCR showed better outcome than conventional CPR. In recent clinical studies, E-CPR also showed the same results. In previous studies, alternative CPR using ECMO was reported to be effective for patients in whom return of spontaneous circulation could not be achieved by conventional CPR [1, 10, 18–20]. In OHCA patients, however, the survival rates reported in the literature are relatively low at 4–28.3 % despite using ECLS (Table 6) [1–4]. The current guidelines for CPR recommend that ECLS be considered in CPR when the patient’s time without blood flow is short [9], and a recent study reported that the 30-day survival rate of refractory OHCA patients was 39.3 % when ECLS support is established within the first 30 min after admission (Table 6) [5].

**Table 6:** Literature review of results of extracorporeal cardiopulmonary resuscitation for out-of-hospital cardiac arrest, and our results

	No. of patients	Survival Rate, n (%)	Neurological Outcome, n (%)	Remark
Kagawa et al. 2010 [1]	39	30 day 5 (13)	CPC 1–2 4 (10)	Time interval from collapse to ECLS start, min (IQR); 59(45–65). Successful percutaneous cannulation (38/39; 97 %). Complications after ECLS; cannula site bleeding (23/39; 59 %), acute kidney injury (9/39; 23 %), leg malperfusion (8/39; 21 %), pneumonia (7/39; 18 %), sepsis (3/39; 8 %).
Le Guen et al. 2011 [2]	42	28 day 2 (4)	CPC 1–2 2 (4)	24 h survival rate after ECLS (17/42; 40 %). 48 h survival rate after ECLS (5/42; 12 %). 28 day survival rate after ECLS (2/42; 4 %).
Avalli et al. 2012 [3]	18	28 day 1(5)	GOS ≥ 4 1 (5)	Survival rate of patients with OHCA (1/18; 5 %) lower than IHCA (11/24; 46 %). Low flow time of OHCA (77 min) longer than IHCA (55 min). Cannulation failure: two in OHCA (2/18; 11 %) In OHCA, complications ECMO; distal leg perfusion (4/18; Haneya22%), femoral leg lesion (2/18; 11 %).
Haneya et al. 2012 [29]	26	Survival discharge 6 (23.1)		Forty patients (47 %) were successfully weaned and 29 patients (34 %) survived to hospital discharge.
Maekawa et al. 2013 [5]	53	3 mon 15 (28.3)	CPC 1 or 2 at 3mon: 8 (15.1)	Survival rate at 3 months (15/53; 28.3 %) Complications after ECLS; cannulation site bleeding (17/52; 32.7 %), cannulation site infection (4/52; 7.7 %). Pupil diameter on hospital arrival may be associated with the neurologic outcome.
Leick et al. 2013 [4]	28	11 (39.3)	No check	Door to ECLS implantation time < 30 min → significantly improved the 30 day survival Complications after ECLS; bleeding (9/28; 32 %), lower limb ischemia (1/28; 3.6 %).
Sakamoto et al. 2014 [30]	260		32 (12.3)	ECPR 26 hospitals vs. non-ECPR 20 hospitals CPC 1–2 at 1 months: 12.3 vs. 1.5 % CPC 1–2 at 6 moths: 11.2 vs. 2.6 %
Stub et al. 2014 [31]	9	Survival discharge 3 (33.3)	CPC 1 or 2 at discharge 3 (33.3)	CHEER trial (11 with OHCA, 15 with IHCA) CPC score 1: 14/26 (54 %) collapse to initiation of ECMO: 56 min
Our results	23	30 day 10 (43.5)	CPC 1: 7 (30) CPC 4: 3 (13)	Door to ECMO team activation time < 10 min for refractory OHCA. Rapid and accurate ECMO implantation in the catheter laboratory. Complications after ECLS; cannulation site bleeding (3/23; 13 %). Oliguria may be a predictor for mortality. Brain CT just after ECMO and following brain imaging may be a predictor for neurologic outcome.

*n* number of patients, *CPC* cerebral performance category, *ECLS* extracorporeal life support, *GOS* glasgow outcome scale, *OHCA* out-of-hospital cardiac arrest, *IHCA* in-hospital cardiac arrest, *ECMO* extracorporeal membrane oxygenation, *CT* computed tomography

Our study found a 30-day survival rate of 43.5 %, which was superior to that of previous studies of 55 min (median) for implanting ECMO after arrival at the emergency department. Our better survival rate may have been due to activation of the ECMO team within 10 min for refractory cardiac arrest. Le Guen et al. [2] and Avalli et al. [3] reported that ECLS was considered in the case of absence of ROSC after 30 min of CPR. Kagawa et al. [1] and Chen et al. [10] suggested that it be considered within 20 min and after 10 min without ROSC, respectively. In the present study, all patients were rapidly and accurately provided ECMO during CPR in the catheterization laboratory near the emergency department; as a result, the incidence of bleeding at the cannulation site was lower than in other studies (Table 6) [1, 3]. However, 3/23 patients (13 %) had bleeding at the cannulation site and all of them died. Nagao et al. [21] reported that bleeding or hematoma at the cannulation site complicates patient management, negatively affecting the outcome.

In the present study, multivariate logistic regression analysis identified a 24 h urine output (UO) of ≤ 0.5 mL · kg-1 · h-1 as an independent risk factor of 30-day mortality. Zhang et al. and Prowle et al. reported that lower urine output is associated with an increase of mortality in critically ill patients. [22, 23] Thus, it is noteworthy that our patients with oliguria in the first 24 h had a significantly different mortality rate, suggesting that oliguria during the first 24 h after ECMO may be a useful predictor of survival.

The neurologic outcome is a grave concern following ECLS [1–4, 11, 24–27]. A potential limitation of a wider use of ECLS in refractory cardiac arrest was the fear that it might lead to severe neurological sequelae in the patients, leading to costly resources and considerable suffering for the patients and their families [28]. However, few studies have analyzed the predictors of neurologic outcome after ECLS. Maekawa et al. [5] suggested that pupil diameter on hospital arrival may be a key predictor of neurologic outcome in patients who received ECLS. However, we observed no difference in survival rate depending on pupil diameter on arrival to the emergency room. Instead, we observed that the pupil reflex in all of the surviving patients who underwent ECMO changed from negative to positive.

Interestingly, in this study, 20/23 patients had a brain CT just after ECMO implantation. According to the results, 6 of the 9 patients (66.7 %) who had a normal CT just after ECMO survived and 3 of them had a normal follow-up CT. The other 3 showed minimal hypoxic brain damage, but all were able to live a normal life. However, only 3 of the 11 patients (27 %) who had hypoxic brain damage on initial brain CT survived; their CPC grade was 4. These results suggest that hypoxic brain damage immediately following ECMO can result in a poor neurologic outcome. Furthermore, all four patients who had intracranial hemorrage due to hypoxic brain damage on follow up images died regardless of the initial CT findings. Based on our results, CT of the brain just after ECMO and follow-up brain images may help to predict the neurologic outcome and survival. However, further studies are necessary to fully validate this finding.

### Study limitation

Several limitations must be considered in the interpretation of our results. First, the total number of enrolled patients was very small. Owing to strict indication for ECPR, the number of case of ECPR was inevitably small. Due to the limitation in the statistical analysis, it is important not to make strong conclusions. Therefore, it is imperative for a well- designed study with large sample size to ensure the outcome and risk factor in ECPR. Second, this was a study of a single institution with an experienced ECMO team and a well-established system. However, not all hospitals have the same type of team and system. Thus, our results should be interpreted with caution, although we highlighted the importance of rapid and accurate ECMO. Third, the study was not a randomized controlled trial, but rather a retrospective analysis of our experience with ECMO for refractory OHCA.

## Conclusions

ECMO can be an appropriate therapeutic option in patients with refractory OHCA. On the basis of our findings, the survival rate can be improved by mobilization of the ECMO team within 10 min for refractory OHCA, and rapid and accurate implantation of ECMO. Oliguria during the first 24 h after ECMO may be an independent predictor of mortality. Furthermore, CT of the brain just after ECMO and subsequent brain imaging may be important predictors of neurologic outcome after ECMO.

## Abbreviations

CI: confidence interval
CPC: cerebral performance category
CPR: cardiopulmonary resuscitation
CT: computed tomography
ECLS: extracorporeal life support
ECMO: extracorporeal membrane oxygenation
GCS: Glasgow coma score
OHCA: out-of-hospital cardiac arrest
ROSC: return of spontaneous circulation
UO: urine output
